# Postharvest Fungicide for Avocado Fruits: Antifungal Efficacy and Peel to Pulp Distribution Kinetics

**DOI:** 10.3390/foods9020124

**Published:** 2020-01-23

**Authors:** Jakob A. Shimshoni, Vijayakumar Bommuraj, Yaira Chen, Roy Sperling, Shimon Barel, Oleg Feygenberg, Dalia Maurer, Noam Alkan

**Affiliations:** 1 Department of Food Quality & Safety, Institute for Postharvest and Food Sciences, Agricultural Research Organization, Volcani Center, Rishon Letzion 7528809, Israel; vijayakumar1669@gmail.com (V.B.); chenyair@volcani.agri.gov.il (Y.C.); 2 Department of Instrumental Analytic, Bilacon GMbH, 13088 Berlin, Germany; roy.sperling@bilacon.de; 3 Kimron Veterinary Institute, Department of Toxicology, Bet Dagan 50250, Israel; Shimonba@moag.gov.il; 4 Department of Postharvest, Institute for Postharvest and Food Sciences, Agricultural Research Organization, Volcani Center, Rishon Letzion 7528809, Israel; fgboleg@volcani.agri.gov.il (O.F.); daliam@volcani.agri.gov.il (D.M.)

**Keywords:** avocado, prochloraz, fludioxonil, peel, pulp, stem-end rot, decay, postharvest, distribution kinetics

## Abstract

Postharvest application of fungicides is commonly applied in order to reduce food loss. Prochloraz is currently the only postharvest fungicide registered in Israel and Europe in avocado fruits. Due to its unfavorable toxicological properties, prochloraz will be banned from the end of 2020 for future postharvest usage and therefore a substitute candidate is urgently warranted. Fludioxonil, a relatively safe, wide spectrum fungicide, is approved in Europe and Israel for postharvest use in various fruits, but not avocado. Hence, fludioxonil has been evaluated in the present study as a potential substitute for prochloraz in avocado. The objectives of the present study were to determine fludioxonil efficacy against common fungal infestations in avocado and distribution kinetics between peel and pulp in comparison to prochloraz. At the same concentration range (75–300 µg/L), fludioxonil was as effective as prochloraz in inhibiting postharvest decay, while in the early season cultivars, suffering mainly from stem-end rot, it exhibited a better decay control than prochloraz. Fludioxonil and prochloraz displayed negligible and undetected pulp levels, respectively, due to low peel penetrability. Taken altogether, fludioxonil was found to be a suitable candidate for replacing prochloraz as a postharvest fungicide in avocado.

## 1. Introduction

In light of the worldwide population growth, a steady increase in the quantity of edible agricultural commodities and reduction of food loss is essential [1]. To address this issue, the global use of pesticides on crops plays a major role [1]. Considering the reported toxic effects of pesticides on humans and animals, it is crucial to monitor pesticide residues in agricultural commodities and animal-derived food products and set protective regulations and measurements, assuring public safety. Avocado represents an important part of fruit production and agricultural trade in Israel [2]. Avocado fruits are consumed worldwide in increasing quantities, thanks to the application of pre- and postharvest fungicides and optimized storage conditions [3]. Avocado fruits are prone to attack by several pests and diseases, predominantly *Colletotrichum gloeosporioides*, *Lasiodiplodia theobromae*, and *Alternaria alternata*; hence, in order to ensure the quality and quantity of avocado fruit, postharvest treatments are common practice worldwide [4]. Prochloraz (*N*-Propyl-*N*-(2-(2,4,6-trichlorophenoxy)ethyl)-1*H*-imidazole-1-carboxamide), a broad-spectrum imidazole postharvest fungicide exerting contact poisoning action, is currently approved in the European Union and Israel as a postharvest fungicide in avocado fruits [5,6]. Since prochloraz major metabolites, namely 2,4,6-trichlorophenol (2,4,6-TCP), 1-propyl-1-[2-(2,4,6 trichlorophenoxy)ethyl]urea (BTS 44595), 3-formyl-1-propyl-1-[2-(2,4,6-trichlorophenoxy)ethyl]urea (BTS 44596) and 2,4,6-trichlorophenol (2,4,6-TCP), are suspected of exerting adverse health effects in humans, prochloraz is determined in food commodities as the parent compound together with its major metabolites (total prochloraz) [7]. The US Environmental Protection Agency (EPA) has defined prochloraz minor metabolite 2,4,6-TCP as a probable human carcinogen (Group B2), being listed as a priority pollutant [8]. Numerous studies have been published on the residue behavior and dietary risk assessment of prochloraz in agricultural commodities and environmental samples [7]. However, a substantial knowledge gap exists regarding the depletion kinetics and distribution of prochloraz and its major metabolites during the storage in peel, pulp, and the avocado fruit. Moreover, in Europe, prochloraz will be banned from the end of 2021 for future postharvest usage and therefore a substitute candidate is urgently warranted. Fludioxonil (4-(2,2-Difluoro-1,3-benzodioxol-4-yl)-1*H*-pyrrole-3-carbonitrile), an active ingredient approved in the European Union and Israel for postharvest use in citrus, mango, and pomegranate fruit, is a wide spectrum fungicide of the phenylpyrrole class [9]. Due to fludioxonil favorable toxicological properties as well as excellent antifungal efficacy against *Penicillium* spp., *Alternaria* spp., *Botrytis* spp., *Lasiodiplodia* spp., and *Rhizopus* spp., it has been evaluated in the present study as a potential substitute for prochloraz [10,11].

The aims of the present study were: (1) to determine the efficacy of fludioxonil at various concentrations against common fungal infestations in avocado fruit in comparison to prochloraz; (2) to determine the fludioxonil residue kinetics and distribution between peel, pulp, and whole fruit in comparison to prochloraz; and (3) to perform a dietary risk assessment of prochloraz and fludioxonil residues in avocado fruit. 

## 2. Methods and Materials

### 2.1. Fungal and Plant Material, Media, and Fungicides 

Uniform and high-quality avocado fruit (*Persea americana*) were harvested—cv. Pinkerton (HaSharon area), cv. Ettinger (Nahshonim and Nahsholim areas), and cv. Reed (Nahshonim areas)—from Israel and transported to the postharvest laboratories at the Agricultural Research Organization, Volcani Center, Israel. 

*Lasiodiplodia theobromae* strain Avo 62 was isolated from rotten “Hass” avocado fruit grown in Israel and used for this study. The purified single spore cultures of *L. theobromae* strain Avo 62 was identified by sequencing the amplicon of the PCR amplification with ITS1 and ITS4 primers and was identified as a stem-end rot (SER) causal agent by Koch’s postulate on avocado fruit [11,12]. The fungi were maintained on potato dextrose agar (PDA, Difco Ltd., Le Pont-de-Claix, France) at 22 °C. 

The following fungicides were used: (1) prochloraz (*N*-Propyl-*N*-(2-(2,4,6-trichlorophenoxy)ethyl)-1*H*-imidazole-1-carboxamide), commercial name: Sportak (Bayer, Leverkusen, Germany), soluble concentrate 450 g/L in water; (2) fludioxonil (4-(2,2-Difluoro-1,3-benzodioxol-4-yl)-1*H*-pyrrole-3-carbonitrile), commercial name: Scholar (Syngenta, Huddersfield, UK), soluble concentrate 230 g/L in water.

### 2.2. Effect of Fungicide Application on Avocado Fruit Inoculated with L. Theobromae

Freshly harvested avocado fruit cv. Pinkerton was disinfected with 1% chlorine for 2 min and rinsed twice with autoclaved water. The fruit stem-ends were removed and the site of injury was disinfected with 70% ethanol. The inoculation was performed by dripping 20 µL of *L. theobromae* conidial aqueous suspension (10^5^ conidia/mL) to completely cover the stem-end. After one day of incubation at 22 °C, the fruits were subsequently sprayed for 30 sec with 150 or 300 µg/L of prochloraz (Sportak) or fludioxonil (Scholar) aqueous solution by utilizing a handheld portable pressure sprayer N-50 (Tabor tools, Beit Rimon, Israel) at a rate of 20 mL spray mixture per 10 kg fruit, according to label instructions and dried for 1 h at room temperature. The control fruit was sprayed with tap water under the same conditions. Each treatment group consisted of 30 fruits. Subsequently, the fruits were incubated at 95% relative humidity (RH) and 22 °C for 7 days and the decay diameter was measured. 

### 2.3. Effect of Postharvest Application on Avocado Fruit Natural Rot

The avocado fruit was sprayed for 30 sec with 150 or 300 µg/L prochloraz (Sportak, Leverkusen, Germany) or fludioxonil (Scholar) solutions by utilizing a handheld portable pressure sprayer N-50 (Tabor tools, Beit Rimon, Israel) at a rate of 20 mL spray mixture per 10 kg fruit, according to label instructions and dried 1 h at room temperature. The control fruit was sprayed with tap water. The experiment was repeated with “Reed” and “Ettinger” avocado fruit in two different seasons. The fruit was then air-dried and stored for 21 days in cold storage (CS, 5 °C) and an additional week at room temperature (20 °C) to simulate the long export and additional shelf life (SL), respectively. Each treatment consisted of four cardboard boxes containing 13 to 16 fruit (52 or 64 fruits per treatment). 

SER (caused by *L. theobromae*) or side decay (caused by *C. gloeosporioides* or *A. alternaria*) disease incidence was calculated as the percentage of decayed fruit in a box. SER or side decay severity and a total percentage of decayed were evaluated for each treatment after CS and SL storage. 

Ripening-related physiological parameters of fruit firmness, yellowing, and bottom-end browning were characterized at harvest after 3 weeks of CS (5 °C) and after an additional 1 week of SL storage at 20 °C (Table S1). Fruit firmness was determined by a penetrometer (LT-Lutron FG-20 KG, Indonesia) with an 11 mm probe at two points along the equatorial line of each fruit (five measurements per treatment at each time point); firmness was determined also by evaluation of all the fruits on a relative scale (1, soft to 10, firm). The percentage of bottom-end browning was evaluated in each cardboard box. At the end of the experiment, 20 fruits were opened and the percentage of internal browning was evaluated. 

### 2.4. Reagents for LC-MS/MS

Acetonitrile, glacial acetic acid, and formic acid (all HPLC grade) were obtained from Sigma-Aldrich (Saint Louis, MO, US). Polymerically bonded ethylenediamine-N-propyl phase (PSA) was purchased from Varian (Palo Alto, CA, USA), while anhydrous magnesium sulfate, sodium chloride, anhydrous ammonium acetate, and C18 were purchased from Sigma-Aldrich (Saint Louis, MO, USA). Aqueous solutions were prepared with ultrapure-water (Milli-Q Plus system; Millipore Corp., Billerica, MA, USA). Prochloraz and its metabolites (2,4,6-trichlorophenol (2,4,6-TCP), 1-propyl-1-[2-(2,4,6 trichlorophenoxy)ethyl]urea (BTS 44595), 3-formyl-1-propyl-1-[2-(2,4,6-trichlorophenoxy)ethyl]urea (BTS 44596)], and fludioxonil were of high purity grade (>99.0%), purchased from Sigma-Aldrich (Saint Louis, MO, USA). Individual stock solutions were prepared at 1000 mg/L in acetonitrile and stored at −20 °C. The working solutions were prepared by carrying out appropriate dilutions of the stock solutions.

### 2.5. Residual Distribution Study Design 

Reed avocados were harvested (as described above) and treated with prochloraz (Sportak^®^; 300 or 150 or 75 mg/L active compound) or fludioxonil (Scholar^®^; 300 or 150 or 75 mg/L active compound) and control (water treatment). The dosages were selected based on the highest commonly applied final active compound concentration (300 mg/L) and its serial dilutions. The prochloraz and fludioxonil treatment groups, consisting each of 6 avocados, were sprayed with a handheld portable pressure sprayer N-50 (Tabor tools, Beit Rimon, Israel) at a rate of 20 mL spray mixture per 10 kg fruit, according to label instructions, and dried at room temperature. Fruits were turned manually halfway through the spraying to ensure complete coverage. Subsequently, three avocados per treatment group (time 0) were peeled and the pulp and peel per fruit were separately weighted (without the kernel). The peels of three avocado fruits were pooled as well as the respective pulps and processed separately. The pooled peels were placed into a laboratory blender (Waring^®^, Knutsford, UK), to which double distilled water (DDW) in a weight ratio of 1:3 was added and homogenized for 1 min. A representative portion of the homogenized peel (10 g) was stored at −20 °C in glass flasks protected from light. The pooled pulps were placed into a high-speed blender (Ninja^®^, Needham, MA, USA), to which DDW in a weight ratio of 1:2 was added and homogenized for 1 min. A representative portion of the homogenized pulp (10 g) was stored at −20 °C in glass flasks protected from light. The remaining fruits were stored in the dark at 5 °C for 21 days, after which three avocado fruits per treatment group were randomly selected and processed as described above (time 21 days). The remaining fruits were immediately transferred into a new storage room and stored in the dark for an additional 7 days at a temperature of 20 °C, after which three avocado fruits per treatment group were processed as described above (time 28 days). The avocado analysis of each treatment group as described above was repeated twice and subsequently subjected to sample preparation for fungicide analysis. Based on the average weight fraction of peel and pulp (kernel excluded), the mean fungicide concentration of the whole fruit (peel and pulp) was calculated according to the following equation: C_whole fruit_ = C_pulp_ × f_pulp_ + C_peel_ × f_peel_, with C_whole fruit_ (mg/kg) being the theoretical mean fungicide concentration in the fruit, C_pulp_ and C_peel_ (mg/kg) being the mean pulp and peel concentrations, respectively, and f_pulp_ and f_peel_ representing the weight fraction of pulp and peel (*w/w*), respectively. 

### 2.6. Sample Preparation

The QuEChERS procedure (AOAC Official Method 2007.01) was applied to the peel and pulp avocado samples. In brief, 10 g of thawed sample was placed in a 50 mL centrifuge tube; 10 mL of 1% acetic acid in acetonitrile were added together with 4 g of MgSO_4_ and 1 g of NaCl and immediately shaken for 1 min. The extract was centrifuged at 5000 rpm for 5 min. For the cleanup step, 5 mL of the upper organic layer was transferred into a 15 mL centrifuge tube, which contained a sorbent mixture composed of 250 mg PSA, 250 mg C18, and 750 mg anhydrous MgSO_4_. The mixture was shaken for 30 s and then centrifuged at 5000 rpm for 5 min. Afterward, 3 mL of the supernatant was transferred into a test tube and evaporated using at 40 °C under a gentle stream of nitrogen. The remaining residue was reconstituted with 0.1 mL of 1% formic acid in a DDW/acetonitrile mixture (1:1). The reconstituted solution was subjected to centrifugation at 13,000 rpm for 5 min and subsequently transferred into an injection vial to be analyzed by LC-MS/MS. 

### 2.7. Liquid Chromatography Tandem-Mass Spectrometry (LC-MS/MS) Analysis

All analyses were performed on ACQUITY UPLC (ACQUITY UPLC, XEVO TQD mass spectrometer; Waters Corp., Milford, MA, USA) equipped with a quaternary pump and membrane degasser. The separation column, Zorbax SB-C18 (2.1 × 150 mm i.d. and 3.5 μm; Agilent Technologies, Santa Clara, CA, USA), was kept at 40 °C. An automatic injector was set to inject 10 μL per sample. The mobile phase components were (A) a 10 mM ammonium acetate solution in water and (B) acetonitrile with 0.1% formic acid. The gradient used was initially set at a flow rate of 0.4 mL/min of 95% mobile phase A for 0.25 min. From 0.25 min to 7 min, a linear gradient was used up to 95% mobile phase B, which was maintained for 1 min. Then, a linear gradient was used to reach 95% mobile phase A, maintained for 1 min. Sample analyses were performed using a triple quadrupole system with positive (prochloraz and metabolites) and negative ESI (fludioxonil) (Table 1). The analytes were monitored and quantified using the multiple reaction monitoring (MRM) mode. Optimization of the MS/MS conditions, identification of the parent and product ions, as well as the selection of the cone and collision voltages, were performed with a direct infusion of their individual standard solutions (Table 1). The Masslynx software was used for the LC-MS/MS system control and data analysis. Multiple reaction monitoring (MRM) transitions, as well as major optimized parameters, are provided in Table 1. 

### 2.8. Statistical Analysis

The data presented for fruit decay is an average and standard error. Significance of fungal inhibition between treatments was determined by one-way ANOVA at each time point with JMP Pro 13.0 statistics software, followed by Tukey multiple-range tests. Differences at *p* ≤ 0.05 were considered significant.

Numerical results presented for the fungicide residual study are mean and standard deviation of the mean. Descriptive statistics were performed using a statistical analysis program (GraphPad Prism version 5.00 for Windows, GraphPad Software, San-Diego, CA, USA). A comparison of mean fungicide concentration between peel and pulp was performed using two-way unrepeated ANOVA (GraphPad Prism version 5.00 for Windows, GraphPad Software, San-Diego, CA, USA). Significant interactions were explored with Bonferroni corrected posthoc tests. The two independent within- and between-group variables were time and matrix (peel and pulp). Statistical analysis was performed at a significance level of *p* ≤ 0.05. Time-dependent changes in peel fungicide concentrations were determined by linear regression *F*-test, utilizing the Excel software (Microsoft Excel 2016), in which the difference of the slope from zero was set as the null hypothesis at a significance level of *p* ≤ 0.05. 

## 3. Results and Discussion

### 3.1. Effect of Postharvest Application of Fungicides on SER Caused by L. Theobromae Inoculation

**Figure 1:**
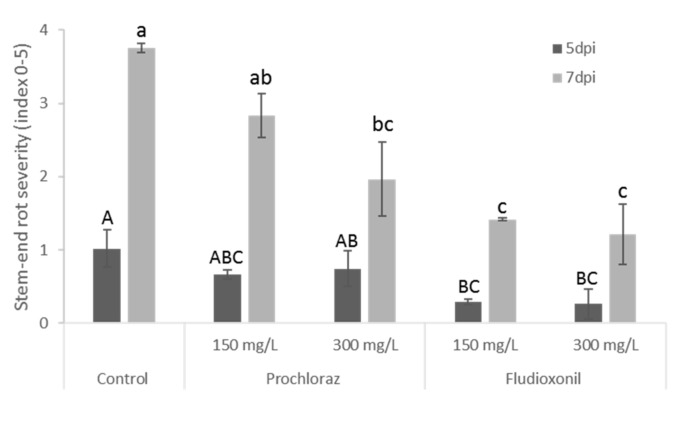
Postharvest fungicide application on “Pinkerton” avocado fruit inoculated with *Lasiodiplodia theobromae*. Stem-end rot severity caused by *L. theobromae* (index 1–10) after 5 and 7 days post-inoculation (dpi). Presented values are average and standard error. Different letters indicate a significant difference (*p* < 0.05) by one-way ANOVA at each time point.

Fludioxonil has been recently demonstrated to be more effective than prochloraz in controlling *Lasiodiplodia* in mango fruit [11]. *Lasiodiplodia* is the main fungi responsible for SER disease and the main concern of postharvest decay in avocado fruit [13,14]. Therefore, conidia of *L. theobromae* were drop-inoculated on the stem-end of ripe Pinkerton avocado fruit. After 24 h, the fruits were treated with fungicides and incubated at room temperature. The application of prochloraz and fludioxonil at all concentrations significantly reduced disease severity (Figure 1). Prochloraz and fludioxonil demonstrated dose-related effective response. Prochloraz at 300 mg/L was as effective as 150 mg/L fludioxonil in controlling avocado fruit SER (Figure 1). The disease incidence was reduced by 71% and 28% in avocado fruit sprayed with 300 mg/L fludioxonil and 300 mg/L prochloraz seven days post-inoculation, respectively (Figure 1). These results indicate that fludioxonil is more effective than prochloraz in controlling SER caused by *Lasiodiplodia* in Pinkerton avocado fruits. This observation is in agreement with the results reported in recent studies, demonstrating fludioxonil superiority over prochloraz in controlling SER caused by *Lasiodiplodia* in mango fruit [13,15,16,17].

### 3.2. Effect of Postharvest Application of Fungicide on Avocado Fruit Natural Rots 

**Figure 2:**
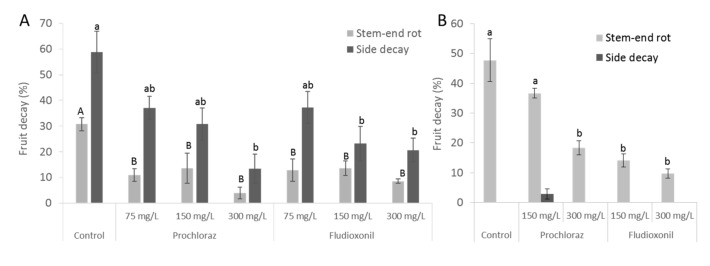
Postharvest fungicide application on avocado fruit and its effect on natural stem-end rot. The fruit was treated with prochloraz or fludioxonil at different concentrations (300, 150, 75 mg/L); then, the fruit was stored at 5 °C for 21 days and for additional shelf life storage period (SL) at 20 °C for 7 days. At the end of the experiment, the stem-end rot and side decay incidence were determined. (**A**) “Reed” fruit from Nahshonim, Israel, 2018. (**B**) “Ettinger” fruit from Magal, Israel, 2015. Presented values are average and SE. Different letters indicate a significant difference (*p* < 0.05) by one-way ANOVA.

In Israel, the early season avocado cultivars (as Ettinger) suffer mostly from SER while the late season cultivars (as Reed) suffer from both anthracnose and SER, but mainly from anthracnose [14]. To evaluate the effect of both fungicides against the natural rots, “Ettinger” and “Reed” avocado cultivars were treated with prochloraz or fludioxonil, followed by CS and SL storage, without any fungal inoculation. Both of the fungicides did not affect the fruit-ripening and quality parameters of softening, internal browning, or bottom-end browning after CS and SL (Table S1) or flavor, which was determined by a small tasting panel at the end of the experiment (data not shown). After CS, the fruits were not ripe and the decay rates were relatively low (Table S2). Following CS and SL storage, 30%–64% of the fruit in the untreated control group displayed SER (Figure 2). Prochloraz showed a reduction of SER occurrence in a concentration-dependent manner (Figure 2). In the Ettinger cultivar, fludioxonil at both concentrations, namely 150 and 300 mg/L, yielded a significant reduction of SER occurrence, comparable to the higher prochloraz concentration of 300 mg/L (Figure 2B). In the “Reed” cultivar, prochloraz and fludioxonil revealed a similar effect in reducing both SER and side decay caused by *C. gloeosporioides* and *A. alternata* (Figure 2A). Our results stand in agreement with a recently published study, in which fludioxonil was found to be more effective against SER than prochloraz in mango fruit while revealing similar efficiency to prochloraz in controlling side decay, caused by both *Alternaria alternata* and *Colletotrichum gloeosporioides* [11]. 

### 3.3. Depletion Kinetics of Prochloraz and its Metabolites in Peel and Pulp

**Figure 3:**
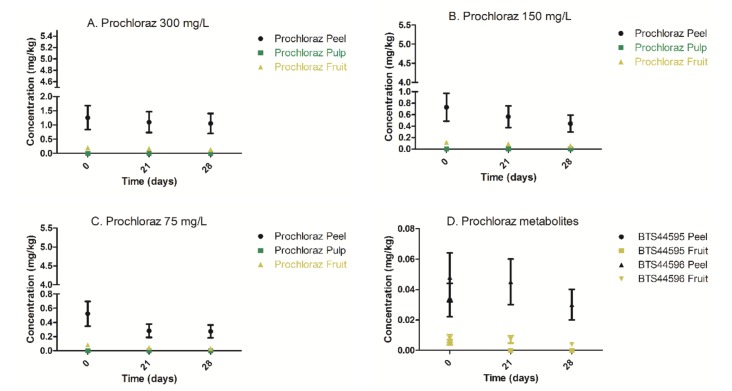
Depletion kinetics of prochloraz and its metabolites (BTS 44595 and BTS 44596) in Reed avocado peel, pulp, and whole fruit following spray application of prochloraz formulations containing 300, 150, and 75 mg/L active compound (Sportak^®^). (**A**) Prochloraz (300 mg/L) depletion in peel, pulp, and whole fruit. (**B**) Prochloraz (150 mg/L) depletion in peel, pulp, and whole fruit. (**C**) Prochloraz (75 mg/L) depletion in peel, pulp, and whole fruit. (**D**) Prochloraz metabolites depletion kinetics in peel and whole fruit, following immersion of Reed avocado in 300 mg/L prochloraz solution. In pulp, no residual levels of metabolites were evident.

Following prochloraz spray application at 300, 150, and 75 mg/L mixture concentrations, no residual levels of prochloraz and its metabolites were found within the pulp (Figure 3, Table 2). Therefore, it is reasonable to conclude that prochloraz could not penetrate the peel under the described experimental conditions. 

Furthermore, kinetic changes of peel prochloraz concentration as a function of storage time were analyzed using the linear regression Fisher test in order to determine whether a significant decline of prochloraz concentration in the peel occurred (Table S3). According to the Fisher test, no statistically significant decline of prochloraz concentrations and its metabolites in the peel at all applied mixture concentrations was observed (Figure 3, Table S3). Consequently, the study’s storage conditions were not sufficient to promote the significant concentration decline/degradation of prochloraz. Notwithstanding, the prochloraz peel concentrations, as well as the calculated whole fruit prochloraz concentrations, were below the current European MRL value (5 mg/kg) during the entire storage period (Figure 3). Hence, the presently recommended postharvest usage of prochloraz at 300 mg/L complies with the European and Israeli MRL value of 5 mg/kg [7]. However, prochloraz is expected to be banned from further postharvest usage by 2021 due to endocrine-disrupting properties and carcinogenic effects observed in animal studies [7]. Since prochloraz MRL value of 5 mg/kg is currently defined for the entire fruit (peel and pulp), we have also calculated the theoretical whole fruit prochloraz concentrations (Figure 3 and Table S4). Naturally, the peel residual levels of postharvest fungicides will always be higher than the corresponding concentrations of the entire whole fruit/vegetable sample due to the external application mode on postharvest agricultural products and the substantial dilution factor contributed by the pulp. The aforementioned calculations of whole fruit levels were based on the average measured peel and pulp weight fractions (kernel excluded), namely f_peel_ (0.16) and f_pulp_ (0.84), obtained from 126 different avocado fruits, as described in Materials and Methods (Section 2.2). As can be seen in Figure 3, the prochloraz concentrations in peel, pulp, and calculated whole fruit were at least three times lower than the current MRL value (5 mg/kg), hence all samples complied with the MRL regulation. Residual levels of the prochloraz metabolites, BTS44595 and BTS44596, were found only on the peel and solely following prochloraz application at the highest mixture concentration of 300 mg/L (Figure 3, Table 2).

The more toxic metabolite, 2,4,6-trichlorophenyl, was undetected in peel and pulp in all samples analyzed during the entire study duration. The metabolites concentration range of BTS44596 in the peel did not change significantly during the entire storage duration and was in the range of 0.048–0.03 mg/kg. However, the metabolite BTS44595 was detectable at time 0 at a concentration of 0.03 mg/kg (Table 2), declining to non-detectable levels after 21 days of storage at 5 °C in the dark. 

Since the avocado peel is considered inedible and unpalatable and is always removed from the fruit before consumption, applying an MRL value for the whole fruit is unreasonable. Moreover, since prochloraz demonstrated endocrine-disrupting properties in animal studies, the currently applied MRL value of 5 mg/kg is exceptionally high as compared to MRL values of other postharvest fungicides of lower toxicity, such as fludioxonil [7,10]. Hence, a reduction of prochloraz MRL in avocado is warranted and can be decreased to a 100 times lower MRL level of 0.05 mg/kg, applied solely for the edible portion of avocado. A reduction of prochloraz MRL will provide an enhanced protective measurement to the end consumer against potential health-related hazards. 

### 3.4. Dietary Risk Assessment of Prochloraz

Currently, for official monitoring purposes, the European Commission, as well as the Israeli Ministry of Agriculture/Health, determines prochloraz and its two major metabolites BTS44595 and BTS44596 in whole avocado fruit (peel and pulp) as total prochloraz [7]. Furthermore, for risk assessment, it was agreed by the European Commission to base the residue definition on the common moiety method and to define the prochloraz residue as the sum of prochloraz and its metabolites containing the 2,4,6-trichlorophenol moiety, expressed as total prochloraz [7]. However, since in the present study, 2,4,6-TCP levels were undetectable (limit of detection 0.002 mg/kg) in peel and pulp and the total calculated metabolite levels of BTS44595 and BTS44596 found in whole avocado fruit samples were below 1% of total prochloraz, we have excluded them from the following dietary risk assessment [7,18].

Prochloraz demonstrated endocrine-disrupting activities in numerous animal studies as well as reproductive and developmental toxicities [7]. In addition, hepatocellular tumors were observed in mice from a dose level of 7.5 mg/kg bw/day [7]. Consequently, in Israel, prochloraz is currently being re-evaluated with the aim of replacing prochloraz with a safer postharvest fungicide. The acceptable daily intake (ADI) of prochloraz is 0.01 mg/kg bw/day, hence 0.6 mg/day for an adult weighing on average 60 kg [7]. The highest calculated prochloraz concentration in the avocado fruit (peel and pulp) was 0.2 mg/kg (Figure 3). Based on the highest concentration found, the average daily intake would be 0.0025 mg/adult (average weight 60 kg) per day based on the average daily avocado consumption of 0.0123 kg/adult/day in Israel [18]. Hence, the estimated prochloraz daily exposure would be 240 times lower than the ADI. Furthermore, since only the pulp is the actual edible part of the avocado, a more realistic daily exposure approach would yield a value of 0.06 µg prochloraz per adult a day, assuming a maximal concentration level equal to the limit of quantitation of 0.005 mg/kg (Table 1). Taken altogether, based on the lack of prochloraz penetrability into the pulp and the sample compliance even at 10 times lower MRL, the regulatory agencies should reconsider banning prochloraz from being further used as a postharvest fungicide in avocado by changing the MRL value accordingly. However, having said that, additional studies are required in order to confirm in prochloraz the lack of peel penetrability in other common avocado varieties such as Ettinger, Hass, and Fuerte, etc., due to their differences in peel thickness and other physicochemical properties. Moreover, other common postharvest fungicide application techniques such as fruit immersion in fungicide mixture solution might yield different results. 

### 3.5. Depletion Kinetics of Fludioxonil in Peel and Pulp

**Figure 4:**
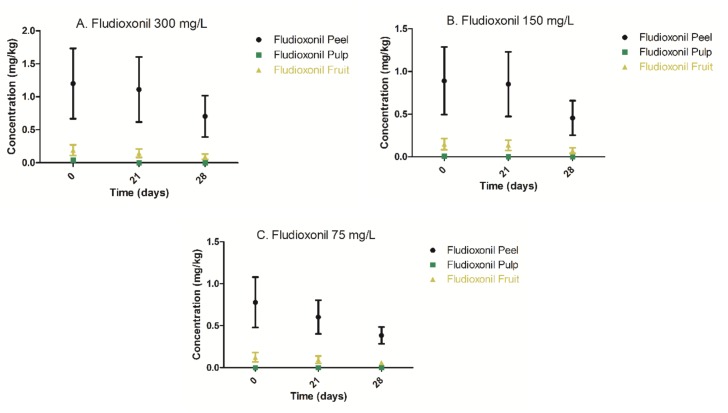
Fludioxonil depletion kinetics in peel, pulp, and whole fruit following spray application of Reed avocado in (**A**) 300 mg/L, (**B**) 150 mg/L, and (**C**) 75 mg/L formulation solution (Scholar^®^). (**A**) Fludioxonil (300 mg/L) depletion in peel, pulp, and whole fruit; (**B**) Fludioxonil (150 mg/L) depletion in peel, pulp, and whole fruit; (**C**) Fludioxonil (75 mg/L) depletion in peel, pulp, and whole fruit.

Fludioxonil application at concentrations of 300 mg/L, 150 mg/L, and 75 mg/L yielded peel residual levels exceeding the fludioxonil European MRL value of 0.4 mg/kg for the avocado at all measured time points but below the US MRL value of 5 mg/kg for avocado. In the pulp, fludioxonil was detectable only at time 0 following spray application of 300 mg/L and 150 mg/L fludioxonil formulations, while at 75 mg/L, no residual levels were detectable (Figure 4). Consequently, the total calculated fludioxonil levels of the whole fruit at all measured time points were at least five times lower than the European MRL value (Figure 4, Table 2 and Table S3). As for prochloraz, no statistically significant decline of fludioxonil concentrations in the peel at all applied mixture concentrations was observed (Figure 4, Table S3). Consequently, under the study’s applied storage conditions, the decline of fludioxonil concentration was negligible (Figure 4, Table 2). The latter observation is not surprising, as fludioxonil is known to be highly stable in the dark, whereas mainly photolysis was shown to play a major role in fludioxonil degradation after foliar application [10]. The time zero measurement is defined as the time point attained immediately after 1 h fruit drying, subsequently to fruit spraying. The occurrence of fludioxonil trace levels in the pulp at time zero (Table 2) is most likely the result of negligible peel penetration, possibly due to a substantial peel to pulp concentration gradient. 

### 3.6. Dietary Risk Assessment of Fludioxonil

Fludioxonil is considered a safe pre- and postharvest fungicide, characterized by the lack of genotoxic, carcinogenic, and teratogenic effects while revealing no scientific evidence of reproductive, developmental, and neurotoxic potential [7]. Hence, fludioxonil is considered markedly a safer postharvest fungicide than prochloraz. Therefore, the relatively low European MRL value of fludioxonil (0.4 mg/kg) in avocado in comparison to prochloraz (MRL = 5 mg/kg) is dubious at best, especially when taking into account fludioxonil’s relatively high ADI of 0.37 mg/kg bw/day as compared to prochloraz ADI of 0.01 mg/kg bw/day [7,10]. 

Following 300 mg/L fludioxonil application, the highest calculated fludioxonil concentration in the avocado fruit (peel and pulp) was 0.3 mg/kg. Consequently, based on the highest fludioxonil concentration determined, the average daily exposure would be 0.0037 mg/adult (average weight 60 kg) per day based on the daily avocado consumption of 0.0123 kg/adult/day in Israel [18]. Hence, the estimated fludioxonil daily exposure would be 100 times lower than its corresponding ADI, thus implying that all the avocado samples treated with fludioxonil were safe for consumption [6,9]. As portrayed for prochloraz in Section 3.2, since only the pulp is the actual edible part of the avocado, and based on the highest pulp concentration found in pulp (0.04 mg/kg), a more realistic daily exposure approach would yield a value of 0.00049 mg fludioxonil per adult a day, being more than 750 times lower than its ADI. Taken altogether, the MRL value of fludioxonil in avocado seems to be arbitrarily too low as compared to other postharvest fungicides displaying less favorable toxicological profiles such as prochloraz. 

## 4. Conclusions

Since prochloraz has been demonstrated to exert endocrine-disrupting effects as well as carcinogenic properties in animal studies, its further usage as a postharvest fungicide will be banned by the European Union by the end of 2021 [19]. Therefore, the fungicidal activity of fludioxonil and its depletion kinetics were tested in avocado fruit in comparison to prochloraz in order to determine its suitability as a prochloraz substitute. The present study demonstrated that fludioxonil was as effective as prochloraz in inhibiting avocado postharvest decay, while in the green and early season cultivars, which suffered mainly from SER caused by *Lasiodiplodia*, it exhibited an even better decay control than prochloraz. Furthermore, fludioxonil and prochloraz displayed only negligible pulp levels, most probably due to low peel penetrability. In addition, according to the calculated whole fruit residual levels, all fruit samples treated with fludioxonil complied with the low European and Israeli MRL value of 0.4 mg/L, indicating its safe usage at concentrations up to 300 mg/L.

## Figures and Tables

**Table 1:** Multiple reaction monitoring (MRM) transition and optimized parameters for pesticides analyzed by LC-MS/MS.

Compound	DP ^1^ (v)	EP ^2^ (v)	CE ^3^ (v)	CXP ^4^ (v)	Q1 ^5^ (g/mol)	Q2 ^6^ (g/mol)	Retention Time (min)	LOD ^7^ mg/kg	LOQ ^8^ mg/kg
Positive ESI ^9^									
(Internal Standard) Bifenthrin D6	37.9	10	27.1	12	312.8	163.9	10.51	0.002	0.005
(Internal Standard) Triphenylphosphat	37.7	10	27.1	12	312.8	163.7	8.41	0.002	0.005
Prochloraz	24	10	17	16	376.0	308.0	8.40	0.002	0.005
Prochloraz-desimidazol-amino	71	10	23	28	327.1	284.0	8.19	0.002	0.005
Prochloraz-desimidazol-formylamino	66	10	21	18	355.0	310.1	8.22	0.002	0.005
Negative ESI									
CAP (Internal Standard)	−65	−10	−24	−11	321.0	152.1	3.50	0.002	0.005
Fludioxonil	−56	−10	−44	−9	247.0	179.9	3.90	0.002	0.005
2,4,6-trichlorophenyl	−45	−10	−32	−7	196.0	159	5.7	0.002	0.005

^1^ DP, declustering potential. ^2^ EP, entrance potential. ^3^ CE, collision energy. ^4^ CXP, collision energy spread. ^5^ Q1, parent ion (molecular ion) used for quantitation and identification. ^6^ Q2, daughter ion (ion fragment of the parent ion) used together with the Q1 for compounds identification. ^7^ LOD, limit of detection. ^8^ LOQ, limit of quantitation. ^9^ ESI, electrospray ionization.

**Table 2:** Statistical comparison analysis of mean residual concentrations (mixed two-way ANOVA) of prochloraz and fludioxonil between peel and pulp of Reed avocado.

Time (Days)	Mean Peel Concentration (mg/kg) ± SD ^a^	Mean Pulp Concentration (mg/kg) ± SD	Statistical Analysis
Prochloraz (300 mg/L)			
0	1.26 ± 0.4	0	*p* < 0.001 (t = 6.2) ^b^
21	1.09 ± 0.3	0	*p* < 0.01 (t = 4.8) ^b^
28	1.05 ± 0.3	0	*p* < 0.05 (t = 3.1) ^b^
Prochloraz (150 mg/L)			
0	0.73 ± 0.24	0	*p* < 0.001 (t = 6.4) ^b^
21	0.56 ± 0.18	0	*p* < 0.001 (t = 4.9) ^b^
28	0.44 ± 0.15	0	*p* < 0.01 (t = 3.9)^b^
Prochloraz (75 mg/L)			
0	0.52 ± 0.17	0	*p* < 0.001 (t = 7.2) ^b^
21	0.28 ± 0.09	0	*p* < 0.01 (t = 3.8) ^b^
28	0.27 ± 0.09	0	*p* < 0.01 (t = 3.7) ^b^
Prochloraz metabolite BTS44595 (after 300 mg/L prochloraz application)			
0	0.03 ± 0.01	0	*p* < 0.001 (t = 9) ^c^
21	0	0	*p* > 0.05
28	0	0	*p* > 0.05
Prochloraz metabolite BTS44596 (after 300 mg/L prochloraz application)			
0	0.046 ± 0.016	0	*p* < 0.001 (t = 5.9)^b^
21	0.045 ± 0.015	0	*p* < 0.001 (t = 5.6) ^b^
28	0.030 ± 0.010	0	*p* < 0.01 (t = 3.7) ^b^
Fludioxonil (300 mg/L)			
0	1.2 ± 0.53	0.04 ± 0.01	*p* < 0.01 (t = 4.4) ^b^
21	1.1 ± 0.49	0	*p* < 0.01 (t = 4.2) ^b^
28	0.7 ± 0.31	0	*p* > 0.05
Fludioxonil (150 mg/L)			
0	0.89 ± 0.39	0.008 ± 0.003	*p* < 0.01 (t = 4.5) ^b^
21	0.85 ± 0.37	0	*p* < 0.01 (t = 4.4) ^b^
28	0.47 ± 0.20	0	*p* > 0.05
Fludioxonil (75 mg/L)			
0	0.78 ± 0.3	0	*p* < 0.001 (t = 5.9) ^b^
21	0.60 ± 0.2	0	*p* < 0.001 (t = 4.6) ^b^
28	0.41 ± 0.1	0	*p* > 0.05

^a^ SD, standard deviation. ^b^ Matrix (peel or pulp) constituted the only significant source of variation (>70% of variation), while time and time X matrix interaction were non-significant. ^c^ Matrix, time, and the interaction of the latter contributed significantly to the source of variation, namely 18.4% (*p* = 0.0002), 36.7% (<0.0001), and 36.7% (<0.0001), respectively. Difference between peel and pulp at *p* ≤ 0.05 was considered as significant.

## Supplementary Materials

The following are available online at https://www.mdpi.com/2304-8158/9/2/124/s1, Table S1: Fruit quality parameters, Table S2: Decay occurrence after cold storage in avocado fruit, Table S3: Linear regression *F*-test of peel pesticide concentration vs. time, Table S4: Calculated total concentrations of prochloraz, its metabolites, and fludioxonil in whole fruit avocado.
