# Supplementary material for: Postharvest Fungicide for Avocado Fruits: Antifungal Efficacy and Peel to Pulp Distribution Kinetics
Source: Foods. 2020 Jan 23;9(2):124. doi: 10.3390/foods9020124 (PMC7074524; doi:10.3390/foods9020124)
Supplement: Supplementary file 1 [file foods-09-00124-s001.pdf]

Table S1: Fruit quality parameters.

Expetimant	Treatment	Concentration (ppm)	Firmness (index 1-10)		Firmness (Newton)			Bottom End Browning (% fruits)	
			CS	SL	T-0	CS	SL	CS	SL
Magal 1.12.15 (Ettinger)	Control		10±0	3.6±0.1	82±4.3	63±3.2	3.9±0.2	1.3±1.3	43.1±6.4
	Prochloraz	150	10±0	3.4±0.2	82±4.3	47±3.9	3.3±0.1	0.0±0	51.0±8.8
		300	10±0	3.0±0.1	82±4.3	50±3.7	3.6±0.2	1.4±1.4	65.9±8.3
	Fludioxonil	150	10±0	3.4±0.2	82±4.3	51±4.0	3.2±0.1	1.3±1.4	54.2±7.2
		300	10±0	3.6±0.2	82±4.3	57±3.3	3.7±0.2	0.0±0	32.5±7.0
Nachshonim 14.4.19 (Reed)	Control		9.8±0.1	1.0±0	138±3.3	137±5.8	7.4±0.3	0.0±0	20.5±4.8
	Prochloraz	75	10.0±0.0	1.0±0	138±3.3	136±3.9	6.4±0.3	0.0±0	28.0±6.4
		150	10.0±0.0	1.0±0	138±3.3	138±1.3	7.0±0.4	0.0±0	24.0±4.3
		300	10.0±0.0	1.0±0	138±3.3	131±5.7	6.4±0.3	0.0±0	20.2±2.1
	Fludioxonil	75	9.9±0.1	1.0±0	138±3.3	122±8.3	6.1±0.3	0.0±0	21.6±4.2
		150	10.0±0.0	1.0±0	138±3.3	128±4.7	5.8±0.4	0.0±0	31.0±8.2
		300	10.0±0.0	1.0±0	138±3.3	141±3.7	7.5±0.4	0.0±0	33.2±7.7

Table S2: Decay occurrence after cold storage in avocado fruit.

	'Ettinger' Magal 2015		'Reed' Nahshonim 2018	
	**Stem end rot (%)**	**Side decay (%)**	**Stem end rot (%)**	**Side decay (%)**
**Control**	0.0+0.0	0.0+0.0	1.9+1.9	0.3+0.2
**Prochloraz 150**	0.0+0.0	0.0+0.0	0.0+0.0	0.0+0.0
**Prochloraz 300**	0.0+0.0	0.0+0.0	0.0+0.0	0.0+0.0
**Fludioxonil 150**	0.0+0.0	0.0+0.0	0.0+0.0	0.0+0.0
**Fludioxonil 300**	0.0+0.0	0.0+0.0	0.0+0.0	0.0+0.0

Table S3. Linear regression F-test of peel pesticide concentration vs time[a].

	Slope Coefficients[b]	R^{2c}	Critical *F value*[d]	P-value[e]
Prochloraz (300 mg/L)	-0.007 ± 0.0016	0.98	50.02	0.09
Prochloraz (150 mg/L)	-0.0096 ± 0.0018	0.96	27	0.12
Prochloraz (75 mg/L)	-0.0094 ± 0.0020	0.95	21.8	0.13
Fludioxonil (300 mg/L)	-0.014 ± 0.011	0.65	1.85	0.40
Fludioxonil (150 mg/L)	-0.012 ± 0.011	0.56	1.28	0.46
Fludioxonil (75 mg/L)	-0.012 ± 0.004	0.88	7.8	0.21

[a]The null hypothesis states, that the linear slope of peel pesticide concentration vs. time equals zero. The F-test enables to determine, whether the slope is significantly different form zero. For calculated p value > 0.05, the linear slope was statistically defined as zero.
[b]Linear slope.
[c]R^2, coefficient of determination, defined as the proportion of the variance in the dependent variable (concentration) that is predictable from the independent variable (time).
[d]F statistics, test statistic for testing the statistical significance of the model.
[e]P-value; when the calculated p value is above the significance level of 0.05, then the null hypothesis is accepted as true, hence, the linear slope equals to zero.

Table S4. Calculated total concentrations of prochloraz, its metabolites and fludioxonil in whole fruit avocado.

Time (days)	Calculated[a] mean whole fruit concentration (mg/kg) ± SD[b]
Prochloraz (300 mg/L)	
0	0.20 ± 0.067
21	0.17 ± 0.058
28	0.14 ± 0.045
Prochloraz (150 mg/L)	
0	0.112 ± 0.038
21	0.09 ± 0.031
28	0.057 ± 0.019
Prochloraz (75 mg/L)	
0	0.083 ± 0.027
21	0.045 ± 0.015
28	0.035 ± 0.014
Prochloraz metabolite BTS44595 (after 300 mg/L prochloraz application)	
0	0.005 ± 0.002
21	0

28	0
Prochloraz metabolite BTS44596 (after 300 mg/L prochloraz application)	
0	0.0076 ± 0.0025
21	0.0072 ± 0.0024
28	0.0050 ± 0.0021
Fludioxonil (300 mg/L)	
0	0.191 ± 0.08
21	0.14 ± 0.064
28	0.092 ± 0.041
Fludioxonil (150 mg/L)	
0	0.15 ± 0.066
21	0.14 ± 0.061
28	0.073 ± 0.032
Fludioxonil (75 mg/L)	
0	0.12 ± 0.05
21	0.09 ± 0.04
28	0.06 ± 0.02

[a] Based on the average weight fraction of peel (0.16) and pulp (0.84) without kernel, the mean fungicide concentration of the whole fruit (peel and pulp) was calculated according to the following equation: $C_{whole\ fruit} = C_{pulp} \times f_{pulp} + C_{peel} \times f_{peel}$; with $C_{whole\ fruit}$ (mg/kg) being the theoretical mean fungicide concentration in the fruit, C_{pulp} and C_{peel} (mg/kg) being the mean pulp and peel concentrations, respectively; f_{pulp} and f_{peel} representing the weight fraction of pulp and peel (w/w), respectively. [b]SD, standard deviation.
